# Association of Quality of Care With Where Veterans Choose to Get Knee Replacement Surgery

**DOI:** 10.1001/jamanetworkopen.2022.33259

**Published:** 2022-09-30

**Authors:** Nicholas J. Giori, Erin E. Beilstein-Wedel, Michael Shwartz, Alex H. S. Harris, Megan E. Vanneman, Todd H. Wagner, Amy K. Rosen

**Affiliations:** 1 Center for Innovation to Implementation, VA Palo Alto Health Care System, Palo Alto, California; 2 Department of Orthopedic Surgery, Stanford University, Redwood City, California; 3 Center for Health Care Organization and Implementation Research, VA Boston Health Care System, Boston, Massachusetts; 4 Department of Surgery, Stanford University, Stanford, California; 5 Informatics, Decision-Enhancement and Analytic Sciences Center (IDEAS), VA Salt Lake City Health Care System, Salt Lake City, Utah; 6 Department of Internal Medicine, University of Utah School of Medicine, Salt Lake City; 7 Department of Population Health Sciences, University of Utah School of Medicine, Salt Lake City; 8 Department of Surgery, Boston University School of Medicine, Boston, Massachusetts

## Abstract

**Importance:**

Recent legislation expanded veterans’ access to Veterans Health Administration (VA)-purchased care. Quality should be considered when choosing where to get total knee arthroplasty (TKA), but currently available quality metrics provide little guidance.

**Objective:**

To determine whether an association exists between the proportion of TKAs performed (vs purchased) at each VA facility and the quality of care provided (as measured by short-term complication rates).

**Design, Setting, and Participants:**

This 3-year cohort study used VA and community care data (fiscal year 2017 to fiscal year 2019) from the VA’s Corporate Data Warehouse. Complications were defined following the Centers for Medicare and Medicaid Services’ methodology. The setting included 140 VA health care facilities performing or purchasing TKAs. Participants included veterans who had 43 371 primary TKA procedures that were either VA-performed or VA-purchased during the study period.

**Exposures:**

Of the 43 371 primary TKA procedures, 18 964 (43.7%) were VA-purchased.

**Main Outcomes and Measures:**

The primary outcome was risk-standardized short-term complication rates of VA-performed or VA-purchased TKAs. The association between the proportion of TKAs performed at each VA facility and quality of VA-performed and VA-purchased care was examined using a regression model. Subgroups were also identified for facilities that had complication rates above or below the overall mean complication rate and for facilities that performed more or less than half of the facility’s TKAs.

**Results:**

Among the study sample’s 41 775 veterans who underwent 43 371 TKAs, 38 725 (89.3%) were male, 6406 (14.8%) were Black, 33 211 (76.6%) were White, and 1367 (3.2%) had other race or ethnicity (including American Indian or Alaska Native, Asian, and Native Hawaiian or other Pacific Islander); mean (SD) age was 66.9 (8.5) years. VA-performed and VA-purchased TKAs had a mean (SD) raw overall short-term complication rate of 2.97% (0.08%). There was no association between the proportion of TKAs performed in VA facilities and risk-standardized complication rates for VA-performed TKAs, and no association for VA-purchased TKAs.

**Conclusions and Relevance:**

In this cohort study, surgical quality did not have an association with where veterans had TKA, possibly because meaningful comparative data are lacking. Reporting local and community risk-standardized complication rates may inform veterans’ decisions and improve care. Combining these data with the proportion of TKAs performed at each site could facilitate administrative decisions on where resources should be allocated to improve care.

## Key Points

**Question:**

Is there an association between the proportion of total knee arthroplasties (TKAs) performed (vs purchased) by Veterans Health Administration (VA) facilities and the quality of care provided?

**Findings:**

This 3-year cohort study of 43 371 TKAs on veterans, using Centers for Medicare and Medicaid Services’ definition of short-term TKA-related complications, found no association between the proportion of TKAs performed in VA facilities and risk-standardized complication rates for VA-performed TKAs, and no association for VA-purchased TKAs.

**Meaning:**

These findings suggest that the VA should monitor and report meaningful measures of surgical quality for VA-performed or purchased TKAs to optimize veteran care and inform quality improvement.

## Teaser

This cohort study examines whether an association exists between the proportion of total knee arthroplasties performed (vs purchased) at each Veterans Health Administration facility and the quality of care provided as measured by short-term complication rates.

## Introduction

The 2014 Veterans Access, Choice and Accountability Act (Choice Act, Public Law 113-146) expanded veterans’ access to community health care paid for by the Veterans Health Administration (VA) (VA-purchased care). The Choice Act gave VA enrollees waiting longer than 30 days, living more than 40 miles from a VA clinic, or experiencing hardship accessing VA care (VA-performed care) the option of seeking care from physicians, hospitals, and health care groups within their communities (hereafter, *community health care facilities*). Thirty-one percent of veterans received VA-purchased care through the Choice program.^1,2^ VA-purchased care availability expanded with the 2018 Maintaining Internal Systems and Strengthening Integrated Outside Networks Act (MISSION Act, Public Law 115-182). These laws were passed primarily to address travel distance or wait time concerns for veterans at their local VA facilities.

Little information exists on the quality of VA-performed and VA-purchased care. Some comparative data are available on the Access and Quality in VA Healthcare website,^3^ but these are limited in scope, lack meaningful specialty-specific metrics, and do not account for population differences. Several studies have shown that VA-performed care compares favorably with community care.^4,5,6,7^ VA-performed surgical procedures, such as total knee arthroplasties (TKAs), have comparable or lower overall rates of complications and readmissions than VA-purchased surgical procedures.^8,9,10^ However, these findings may obscure quality differences across VA facilities and across community health care facilities from whom the VA purchases care.

The TKA-related findings are noteworthy for several reasons. First, TKAs are the most common inpatient surgery performed by the VA, requiring the full spectrum of personnel, services, and facilities needed to support quality surgical care. Second, because TKAs are largely elective, Choice- and/or MISSION-eligible veterans with advanced knee arthritis can choose between getting VA-performed or VA-purchased care.

The lack of meaningful VA and community quality data are a problem that should be rectified if these data could help veterans make better choices about where to receive care. To investigate this, we built on prior work by Harris et al (2021)^9^ to evaluate, at the facility level, whether the proportion of veterans having VA-performed (vs VA-purchased) TKAs was associated with the quality of TKAs (as measured by standardized short-term complication rates) performed or purchased by that VA facility. If veterans selected high-quality care without access to meaningful comparative quality information, then there is no need to develop and publish such metrics. If instead, veterans did not preferentially select high-quality care, then such metrics may be needed to better inform choices and improve overall care.

## Methods

This study was reviewed by VA Palo Alto, Salt Lake City, and Boston Research and Development Committees and by the Stanford University and University of Utah institutional review boards. It was approved as a quality improvement project and granted waiver of informed consent because it was deemed not to be human participants research. This study followed the Strengthening the Reporting of Observational Studies in Epidemiology (STROBE) reporting guideline for cohort studies.

### Study Data

We identified VA-performed and VA-purchased elective TKAs done between October 1, 2016, and September 30, 2019, using VA and community care data from the VA’s Corporate Data Warehouse. Inpatient TKAs were identified by CPT code 27447 (total knee arthroplasty). Per Centers for Medicare and Medicaid Services (CMS) methodology, a veteran could contribute only 1 randomly selected TKA per fiscal year. TKAs were associated with the VA facility that either performed or purchased the TKA.

### Population and Sample

Similar to Harris et al (2021),^9^ the population included VA-performed and VA-purchased unilateral TKAs (n = 50 764) in 48 928 veterans from 140 VA facilities. We excluded outpatient, nonelective, and bilateral TKAs. This yielded 41 775 veterans at 140 facilities who had 43 371 TKAs (Table 1). Bilateral TKAs (15 VA-performed; 434 VA-purchased) were excluded due to their greater complexity and risk.

**Table 1.** TKA Case Inclusion

TKAs	VA-performed	VA-purchased
Inpatient TKAs identified (excluding outpatient), No.	27 689	23 911
Unilateral only, No. (No. removed)	27 462 (227)	23 302 (609)
TKAs without CMS exclusions, No. (No. removed)	25 388 (2074)	19 982 (3320)
TKAs randomly selected–one per FY per patient, No. (No. removed)	24 407 (981)	18 964 (1018)
Final No. of TKAs included	24 407	18 964

### Variables in Risk-Adjustment Model

Post-TKA complications were identified following the 2019 CMS TKA Complication Measure. These include complications related to acute myocardial infarction, mechanical dysfunction, joint or wound infection, pneumonia, pulmonary embolism, sepsis or septic shock, and bleeding.^11^ The primary outcome was any surgical complication (no or yes).^9^

Sociodemographic variables included age, sex, race, marital status, rurality, priority level, and Nosos risk score. Race information was originally self-reported by the veteran and was obtained from the electronic health record. Race was included to ascertain whether there were any racial differences in patients choosing to seek care outside of the VA health care system. Priority level indicates a veteran’s severity of service-connected disabilities and income level.^12^ The Nosos score was developed to characterize disease burden of Veterans for the purpose of projecting total VA costs.^13,14^ The Nosos score and individual comorbidities known to be associated with a higher risk of complication (hypertension, hyperlipidemia, anemia, deep vein thrombosis, and smoking status) were included in the risk adjustment model.

A dichotomous VA-purchased vs VA-performed variable was the key independent variable. Although a VA facility may have purchased TKAs from multiple health care facilities, they were aggregated into 1 entity for each facility as most individual community health care facilities performed too few TKAs to analyze individually.

Consistent with the CMS approach to facility profiling, we did not include facility-level variables in the risk adjustment model. However, we did examine the association between facility surgical complexity (complex, intermediate, standard, or ambulatory/no surgical complexity^15^) and (1) proportion of TKAs performed and (2) the complication rate.

### Statistical Analysis

Age, sex, marital status, rurality, Nosos risk score, comorbidities, and operative complexity level of the local VA facility were compared between patients who had VA-performed or VA-purchased TKAs using *t* tests or χ^2^ tests as appropriate with statistical significance defined as *P* < .05; we also calculated effect sizes (ie, standardized mean differences [SMD]). We interpreted effect sizes as small if they were greater than 0.20 and medium if they were greater than 0.50.^16^

We used a mixed-effects logistic regression model with dependent variable *presence or absence of a complication*, as described in Harris et al.^9^ Whereas Harris’ findings were based on analysis of the random effects (ie, conditional estimates), we calculated marginal results (relevant for conclusions about subgroups or populations) using an extension of the approach used by CMS in hospital profiling. Specifically, we calculated risk-standardized complication rates and used bootstrapping to evaluate statistical significance (see eAppendix in the Supplement for further details and Krumholz et al^17^).

Importantly, each facility’s VA-purchased and VA-performed TKA random effects are shrunken estimates^18^ calculated by giving some weight to the facility mean and some to the overall mean across all facilities. In facilities with fewer observations, more weight is given to the overall mean, which shifts its standardized complication rate closer to the average complication rate. In larger facilities where there is little shrinkage, the facility-level complication rates will be closer to the observed rate and thus more dispersed around the overall average.

We used scatter plots to portray the proportion of TKA performed by each facility and the short-term complication rate at that facility, and a regression model to examine whether there was statistical significance. Statistical significance was defined as *P* < .05. Four subgroups were defined based on whether the facility had a complication rate above or below the mean raw overall complication rate and whether the facility performed more or less than half the TKAs from that facility. We ran linear regression models with dependent variables: (1) the proportion of TKAs performed at the facility and (2) the standardized complication rates. Independent variables were those in the risk adjustment model plus facility surgical complexity level. Complexity level was coded into 3 categorical (coded 0 or 1) independent variables (complex was the reference group) and, in a second run of the model, coded as a single variable with values from 1 (complex) to 4.

## Results

We identified a total of 43 371 primary TKA procedures: 24 407 (56%) were VA-performed and 18 964 (44%) were VA-purchased. Among the 41 775 veterans included in the study, 38 725 (89.3%) were male, 6406 (14.8%) were Black, 33 211 (76.6%) were White, and 1367 (3.2%) had other race or ethnicity (including American Indian or Alaska Native, Asian, and Native Hawaiian or other Pacific Islander); mean (SD) age was 66.9 (8.5) years. Additional sociodemographic characteristics are summarized in Table 2. Due primarily to large sample sizes, there were statistically significant differences in nearly all measured characteristics between Veterans having VA-performed vs VA-purchased TKAs. Results from the standardized mean difference (SMD) comparison revealed differences between the 2 groups on percentage urban (SMD = 0.384 [95% CI, 0.37-0.41]), unknown rurality (SMD = 0.432 [95% CI, 0.43-0.47]), Nosos risk score (SMD = 0.950 [95% CI, 0.91-0.95]), hypertension (SMD = 0.390 [95% CI, 0.37-0.41]), and hyperlipidemia (SMD = 0.209 [95% CI, 0.19-0.23]). Ambulatory surgery centers (ASCs) and facilities classified as having no operative complexity had a larger proportion of purchased TKAs than performed TKAs (SMD = 0.771 [95% CI, 0.75-0.79]); a difference was also found for standard operative complexity facilities (SMD = 0.450 [95% CI, 0.43-0.47]). Facilities rated as complex in their operating capacity had more performed TKAs than purchased TKAs (SMD = 0.772 [95% CI, 0.75-0.79]).

**Table 2.** Demographics and Comorbidities of the Patients in This Study

Characteristic	Patients, No. (%)			*P* value	SMD (95% CI)
	Overall (N = 43 371)	VA-purchased (n = 18 964)	VA-performed (n = 24 407)		
Age, mean (SD)	66.89 (8.51)	66.36 (8.86)	67.30 (8.20)	<.001	0.110 (0.09-0.13)
Sex					
Male	38 725 (89.3)	16 334 (86.1)	22 391 (91.7)	<.001	0.006 (0.01-0.03)
Female	4646 (10.7)	2630 (13.9)	2016 (8.3)	<.001	0.006 (0.01-0.03)
Race					
Black	6406 (14.8)	2555 (13.5)	3851 (15.8)	<.001	0.065 (0.05-0.09)
White	33 211 (76.6)	14 414 (76.0)	18 797 (77.0)	.014	0.024 (0-0.04)
Other^a^	1367 (3.2)	688 (3.6)	679 (2.8)	<.001	0.048 (0.03-0.07)
Not known	2387 (5.5)	1307 (6.9)	1080 (4.4)	<.001	0.107 (0.09-0.13)
Marital status					
Single	4239 (9.8)	1629 (8.6)	2610 (10.7)	<.001	0.071 (0.06-0.09)
Married	26 284 (60.6)	11 804 (62.2)	14 480 (59.3)	<.001	0.060 (0.04-0.08)
Divorced/separated	10 449 (24.1)	4420 (23.3)	6029 (24.7)	.001	0.033 (0.01-0.05)
Widowed	1910 (4.4)	740 (3.9)	1170 (4.8)	<.001	0.044 (0.03-0.06)
Other/unknown	489 (1.1)	371 (2.0)	118 (0.5)	<.001	0.134 (0.12-0.16)
Rurality					
Rural	17 700 (40.8)	8447 (44.5)	9253 (37.9)	<.001	0.149 (0.14-0.17)
Urban	22 872 (52.7)	7885 (41.6)	14 987 (61.4)	<.001	0.384 (0.37-0.41)
Not known	2799 (6.5)	2632 (13.9)	167 (0.7)	<.001	0.432 (0.43-0.47)
VA Priority group, No. (%)					
Groups 1 and 2	23 687 (54.6)	10 696 (56.4)	12 991 (53.2)	<.001	0.064 (0.05-0.08)
Group 3	5298 (12.2)	2364 (12.5)	2934 (12.0)	.161	0.014 (0.01-0.03)
Groups 4 to 8	14 386 (33.2)	5904 (31.1)	8482 (34.8)	<.001	0.077 (0.06-0.1)
Concurrent Nosos Risk Score, mean (SD)	2.45 (2.09)	1.45 (1.55)	3.21 (2.12)	<.001	0.950 (0.91-0.95)
Comorbidities					
Hypertension	16 164 (37.3)	9052 (47.7)	7112 (29.1)	<.001	0.390 (0.37-0.41)
Hyperlipidemia	10 356 (23.9)	5474 (28.9)	4882 (20.0)	<.001	0.209 (0.19-0.23)
Anemia	2942 (6.8)	1649 (8.7)	1293 (5.3)	<.001	0.135 (0.12-0.15)
Deep vein thrombosis	215 (0.5)	128 (0.7)	87 (0.4)	<.001	0.044 (0.03-0.06)
Smoking history	22 120 (51.0)	9164 (48.3)	12 956 (53.1)	<.001	0.095 (0.08-0.11)
Operative complexity					
ASC or no surgery	4350 (10.0)	4350 (22.9)	0	<.001	0.771 (0.75-0.79)
Complex	25 860 (59.6)	7508 (39.6)	18 352 (75.2)	<.001	0.772 (0.75-0.79)
Intermediate	9805 (22.6)	4331 (22.8)	5474 (22.4)	.317	0.010 (0-0.03)
Standard	3356 (7.7)	2775 (14.6)	581 (2.4)	<.001	0.450 (0.43-0.47)

Abbreviations: ASC, ambulatory surgery center; SMD, standardized mean difference; VA, Veterans Health Administration.

^a^ Other includes American Indian or Alaska Native, Asian, and Native Hawaiian or other Pacific Islander

**Figure 1.**
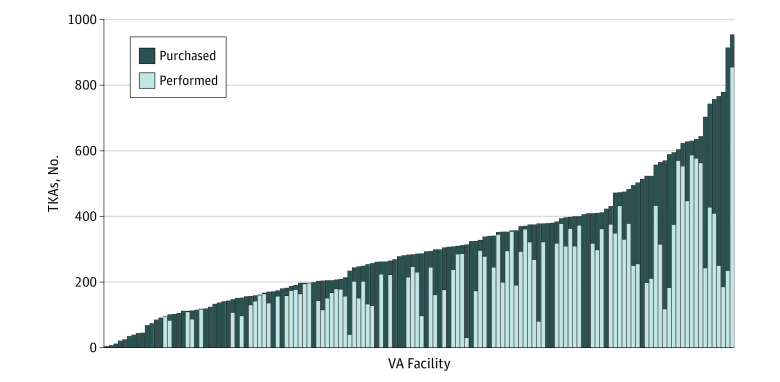
Number of Total Knee Arthroplasties (TKAs) Purchased or Performed A bar graph of TKAs performed and purchased at each facility in the VA health care system in a 3-year period is shown. At each facility, the colors in the bar graph show how many TKAs were performed in a VA facility (light blue), and how many were purchased by the facility in the community (dark blue). VA indicates Veterans Health Administration.

The number of TKAs performed or purchased by each facility ranged from 0 to nearly 1000 in the 3-year study period. Similarly, the proportion of TKAs performed at each VA facility varied widely, with a range from 0% to 100% (Figure 1).

**Figure 2.**
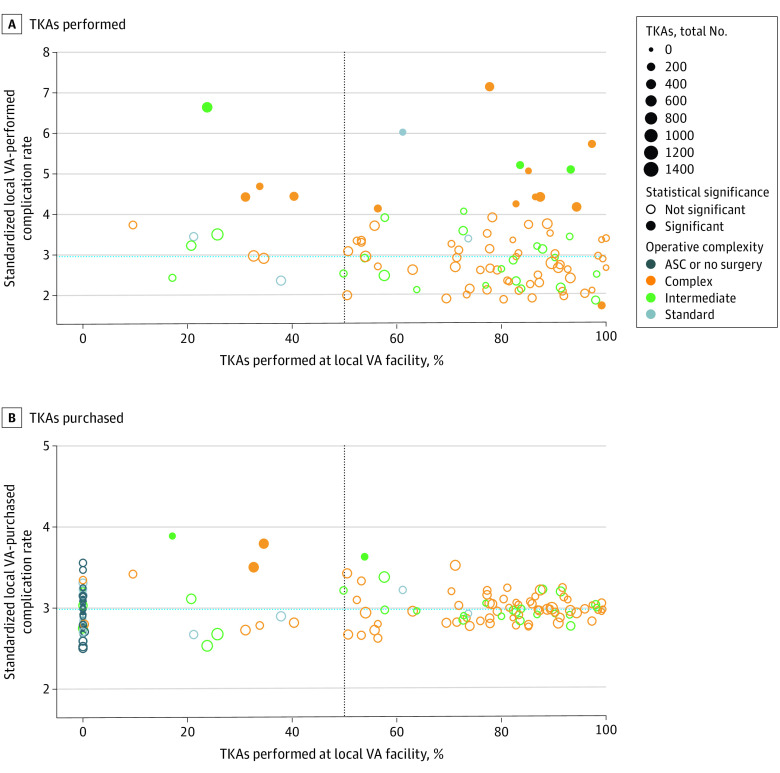
Fully Adjusted Standardized Complication Rates for Total Knee Arthroplasties (TKAs) Performed or Purchased by Individual Veterans Health Administration (VA) Facilities Fully adjusted standardized complication rates (adjusted for patient comorbidities and sociodemographic variables) for TKAs performed (A) and purchased (B) by individual VA facilities are plotted against the percentage of TKAs performed at each local VA facility. The open circles in these graphs indicate no differences in complication rate with respect to the raw overall complication rate. The closed dots represent facilities where there is a statistically significant difference in the VA-performed (Figure 2A) and VA-purchased (Figure 2B) TKA complications when compared with the raw overall complication rate. The size of the dot reflects the total volume of TKA either performed or purchased by that facility, and operative complexity of the facility is denoted by the color of the dot. The finely dotted horizontal line denotes the raw overall complication rate across all patients. Centers appearing above the finely dotted horizontal line had standardized complication rates greater than the overall average, and centers appearing below the finely dotted horizontal line had standardized complication rates lower than the overall average. The vertical line located at 50% on the horizontal axis separates facilities that performed a majority of their TKAs (to the right of the line) from facilities that purchased a majority of their TKAs (to the left of the line). ASC indicates ambulatory surgery center.

Figures 2A and 2B show the VA-performed and VA-purchased standardized TKA complication rates for each facility and the proportion of VA-performed TKAs at that facility. The observed mean (SD) overall complication rate was 2.97% (0.08%), which is displayed as a horizontal line in Figures 2A and 2B. The closeness of many of the VA-purchased care standardized complication rates to the observed overall complication rate reflects the substantial shrinkage in these rates due to small sample sizes. The proportions of individual complications in this cohort have been previously reported by Harris et al.^9^

There was no association between the proportion of TKAs performed in the VA and standardized complication rates for TKAs performed by each VA facility (coefficient = −4.059; 95% CI, −8.263 to 0.145; *P* = .06) (model 1, Table 3). There was no association between the proportion of TKAs performed in the VA and risk-standardized complication rates for TKAs purchased by each facility (model 2, Table 3). The standardized complication rate was not associated with operative complexity (when measured continuously or categorically) (models 5 and 6, Table 3). However, we found that operative complexity was associated with the proportion of TKAs performed in the VA (coefficient = 0.215; 95% CI, 95% CI 0.218-0.284; *P* < .001), with statistically significant differences between complex facilities (model 3) and others (model 4) (Table 3).

**Table 3.** Linear Regression Results

Dependent variable	Percentage TKA performed				Risk-adjusted standardized VA complication rate	
	Model 1	Model 2	Model 3	Model 4	Model 5	Model 6
VA-performed TKA risk-standardized complication rates	–4.059 (2.145)	NA	NA	NA	NA	NA
* P* value	.06	NA	NA	NA	NA	NA
VA-purchased TKA risk-standardized complication rates	NA	3.009 (13.801)	NA	NA	NA	NA
* P* value	NA	.83	NA	NA	NA	NA
Operative complexity continuous	NA	NA	0.215 (0.017)	NA	–0.002 (0.002)	NA
* P* value	NA	NA	<.001	NA	.32	NA
Operative complexity (Reference = complex)	NA	NA	NA	NA	NA	NA
Intermediate	NA	NA	NA	–0.115 (0.052)	NA	0.001 (0.002)
* P* value	NA	NA	NA	.03	NA	*P* = .79
Standard	NA	NA	NA	–0.576 (0.073)	NA	0.007 (0.005)
* P* value	NA	NA	NA	<.001	NA	.20
ASC or no surgery	NA	NA	NA	–0.737 (0.050)	NA	NA
* P* value	NA	NA	NA	<.001	NA	NA
Constant	0.860 (0.071)	0.405 (0.413)	NA	0.0001 (0.042)	NA	0.031 (0.001)
*P* value	<.001	.33	NA	>.99	NA	<.001
Observations	96	138	140	140	96	96
*R^2^*	0.037	0.0003	0.621	0.646	0.011	0.018
Adjusted *R^2^*	0.026	–0.007	0.618	0.638	0.0002	–0.003
Residual standard error	0.218 (*df* = 94)	0.386 (*df* = 136)	0.239 (*df* = 138)	0.233 (*df* = 136)	0.010 (*df* = 94)	0.010 (*df* = 93)
*F* statistic	3.582 (*df* = 1; 94)	0.048 (*df* = 1; 136)	226.268 (*df* = 1; 138)	82.607 (*df* = 3; 136)	1.015 (*df* = 1; 94)	0.849 (*df* = 2; 93)

Abbreviations: ASC, ambulatory surgery care; TKA, total knee arthroplasty; NA, not applicable; VA, Veterans Health Administration.

VA facilities occupied all 4 quadrants of Figures 2A and 2B. In the lower right quadrant of Figure 2A, 45 VA facilities performed more than half of their TKAs. Each of these facilities also had a standardized complication rate for VA-performed TKAs that was below the observed overall complication rate. For 1 VA facility, this difference was statistically significant (solid dot in the lower right quadrant of Figure 2A [point value: 1.7 = 95% CI, 0.63%-2.87%]).

In the lower left quadrant of Figure 2A, we identified 4 VA facilities that purchased more than half of their TKAs. They each also had complication rates for VA-performed TKAs that were below the overall complication rate, although none of these differences were statistically significant. In the upper right quadrant, 38 VA facilities performed most of the TKAs, but their complication rates were higher than the raw overall complication rate. For 11 of these VA facilities, these differences were statistically significant (eg, one facility point estimate was 4.15% [95% CI, 2.98% -5.31%], another facility point estimate was 6.03% [95% CI, 5.04%-7.02%]). In the upper left quadrant, 9 VA facilities had complication rates that were higher than the overall complication rate, but most TKAs from that facility were purchased. In 4 VA facilities, the difference in complication rates was statistically significant (for example, one facility point estimate was 4.43% [95% CI, 3.38%-5.48%], another facility point estimate was 6.64% [95% CI, 5.81%-7.50%]).

Of note, 42 facilities purchased all their TKAs, and 2 facilities performed only a single TKA. They were not included in Figure 2A because there were no VA complication rates to plot on the vertical axis; however, they appear at the far left of Figure 2B. They constituted one-fifth group of VA facilities.

Despite the substantial shrinkage that occurred when calculating VA-purchased standardized complication rates, there were 4 VA facilities that purchased TKA care from community health facilities that had statistically significantly higher complication rates than the raw overall complication rate (for example, one facility point estimate was 3.88% [95% CI, 3.29%-4.46%], another facility point estimate was 3.50% [95% CI, 2.99%-3.99%]) (Figure 2B).

## Discussion

The Choice and MISSION Acts of 2014 and 2018 increased veterans’ access to VA-purchased community care primarily to address wait times and travel distance. However, veterans’ decisions to seek VA-performed or VA-purchased care can be complex and multifactorial. Regarding wait times, research has shown that for orthopedic clinic appointments, VA-purchased care had comparable wait times to VA-performed care in both rural and urban settings.^19^ Regarding travel distance, it is notable that for cataract surgery (which is also elective), 26% of VA-purchased procedures occurred in facilities further from the patient’s home than the closest VA facility performing cataract surgery.^20^ Quality of care should be an important consideration, but we found that the proportion of TKA cases from VA facilities that were VA-performed was not associated with quality of VA-performed TKAs, and also not associated with quality of VA-purchased TKAs. This may not be surprising as specific and relevant surgical quality metrics for TKAs are not currently available. There is thus a need for better publicly reported data, similar to what is presented here, to educate veterans and the clinicians who care for them as this may improve overall veteran care. Although the percentage of TKAs performed by the VA facility and the VA-performed risk-standardized complication rate were not statistically associated, the *P* value was .06, suggesting that continued monitoring may be important as more data become available.

We were intrigued by the other observations made when graphically displaying the results shown in Figure 2, and the potential that these could have in guiding local patient care decisions, policy, or resource allocation. We suggest the following possible interpretations. At VA facilities that performed better-than-average quality TKAs and performed more than half of the TKAs at that facility, veterans and referring clinicians should be informed of the high quality of care provided at the VA facility. These high-performing facilities should continue to receive adequate resources to sustain their good performance. Best practices at these facilities could be used to inform quality improvements at VA facilities that reside in other quadrants.

At VA facilities that performed high-quality TKAs but still purchased most of their TKAs in the community, the reasons for the high community referral rate should be explored, particularly if these facilities were in locations demanding a high volume of TKA care (large dots in Figure 2). Patients and referring clinicians should similarly be informed of the high quality of care provided at these VA facilities. It may be that additional resources could be allocated to expand the number of high-quality TKAs performed at that local facility, thus improving overall veteran care.

Facilities that appeared to have quality issues and performed more than half of the TKAs at that facility may require further attention. Local comparisons of various measures of quality with regional VA-purchased options are needed to further investigate this concern. If the option for VA-purchased care provides higher quality surgical procedures, then the immediate solution would be to increase community care utilization. Longer term, because capacity to perform TKA surgery is apparently not a concern, an assessment should be made regarding whether the local VA facility’s quality issue can be addressed and remedied.

At facilities that performed less than half of all TKAs at their facility and also had higher short-term complication rates than the overall mean complication rate, greater utilization of community care may be appropriate in the short term, assuming that the quality of locally available VA-purchased TKAs is higher than that provided by the specific VA facility. An assessment of the potential costs and benefits could be conducted to determine whether it is worth the substantial investment needed to improve both capacity and quality of lower-performing VA facilities or to outsource TKA care to the community (where quality may not be known).

For facilities that do not currently perform TKAs and purchase all TKA surgical procedures, starting a TKA surgical program would involve a large investment in resources and personnel. Such a commitment would need to be driven by a high demand for the service in the area as well as few or poor community options.

Finally, we identified several VA facilities that were purchasing TKAs with lower-than-average quality. This should be investigated and adjustments should be made, possibly using higher-performing local or regional VA medical centers or other community options.

### Limitations

This study has several limitations. First, short-term complications are only one aspect of quality. Longer-term complications, patient satisfaction, function, or pain relief are other possible measures of quality, although data on such measures is often difficult to obtain. Second, we assessed only specific major complications rather than more common minor complications. Third, each VA facility usually purchased TKA care from more than one community health facility. Given the small number of cases seen by individual community health care facilities, it was not possible to calculate statistically meaningful comparisons to differentiate the quality of each community health care facility. Even when we combined individual community facilities into an aggregate community facility for a facility, there was substantial shrinkage which made it difficult to determine if the quality of care purchased by each facility was higher or lower than the overall mean complication rate. Additional work is needed to best estimate the performance of individual community facilities when sample sizes are small. Fourth, it is possible that there are differences in coding practices between VA and community facilities given the link between coding and billing.^21^ This could have introduced systemic bias in our results, although major complications are likely to be coded similarly across institutions. Fifth, our findings are specific to the period we studied and may not reflect current practice. Sixth, we only evaluated results of inpatient TKAs. During our study period, TKAs were primarily performed in the inpatient setting, although practice has begun to shift more recently. Seventh, bilateral TKAs were excluded from this analysis because they are generally done as a part of a larger operation with a higher risk, and because patients who are selected to have bilateral TKAs have a different disease burden.^22^ Although a limitation, they represented less than 0.1% and 2% of all VA-performed and VA-purchased TKAs, respectively. Finally, although we controlled for risk of complications, unobserved factors not accounted for in our risk adjustment method may have affected the results.

## Conclusions

Surgical quality did not have a statistically significant association with where veterans had TKA, possibly because meaningful surgery-specific comparative data are lacking. Making meaningful quality metrics available, such as local and community risk-standardized complication rates, may help veterans make decisions on where to seek care and thus improve overall care. Combining these data with the proportion of TKAs performed at each site could facilitate administrative decisions on where resources should be allocated to improve care.

## Notes

Supplement. **eAppendix.** Extension of the CMS Risk Adjustment Profiling Model to Include VA-Purchased Care
