# Supplementary material for: Association of Quality of Care With Where Veterans Choose to Get Knee Replacement Surgery
Source: JAMA Netw Open. 2022 Sep 30;5(9):e2233259. doi: 10.1001/jamanetworkopen.2022.33259 (PMC9526089; doi:10.1001/jamanetworkopen.2022.33259)
Supplement: Supplement. — eAppendix. Extension of the CMS Risk Adjustment Profiling Model to Include VA-Purchased Care [file jamanetwopen-e2233259-s001.pdf]

Supplemental Online Content

Giori NJ, Beilstein-Wedel EE, Shwartz M, et al. Association of quality of care with where veterans choose to get knee replacement surgery. *JAMA Netw Open.* 2022;5(9):e2233259. doi:10.1001/jamanetworkopen.2022.33259

eAppendix. Extension of the CMS Risk Adjustment Profiling Model to Include VA-Purchased Care

This supplemental material has been provided by the authors to give readers additional information about their work.

© 2022 Giori NJ et al. *JAMA Network Open.*

eAppendix: **Extension of the CMS risk adjustment profiling model to include VA-purchased care**

In addition to the patient level covariates, we included in the risk adjustment model a fixed effect indicator variable for VA-performed (variable =0) vs. VA-purchased care (variable =1) care. The intercept term was specified as a random effect (for VA-performed care). The (purchased/performed) indicator variable was included a second time in the model, but with a random coefficient (an estimate of the VA-purchased random effect).

Marginal estimates of the standardized complication rates were calculated as follows: 1) For each Veteran, we calculated the predicted probability of a complication from the regression model; 2) For each Veteran, we calculated the expected probability of a complication under the assumption the Veteran had TKA performed at an average VA facility (the VA-performed random effect was set to zero) or purchased by the VA from a community provider with the same complication rate as the average VA facility (the VA-purchased random effects were set to 0); 3) For each VA facility, we calculated the ratio of the sum of the predicteds to the sum of the expecteds for VA-performed and VA-purchased TKAs; and 4) finally, the standardized complication rate at each VA facility for VA-performed and VA-purchased TKA was calculated by multiplying the ratio obtained in step 3 by the overall observed complication rate. Of note, if the ratio in step 3) equals 1, the standardized complication rate equals the overall observed complication rate. To determine statistical significance, we used the cluster bootstrap sampling method used by CMS in many of its performance analyses [17]. However, the risk adjustment model used in the bootstrapping incorporated the VA-purchased random effects, as described above.

© 2022 Giori NJ et al. *JAMA Network Open.*
